# Covalency between the uranyl ion and di­thio­phosphinate by sulfur *K*-edge X-ray absorption spectroscopy and density functional theory

**DOI:** 10.1107/S160057752101198X

**Published:** 2022-01-01

**Authors:** Yusheng Zhang, Wuhua Duan, Qiang Wang, Lei Zheng, Jianchen Wang, Jing Chen, Taoxiang Sun

**Affiliations:** a Institute of Nuclear and New Energy Technology, Tsinghua University, Beijing 100084, People’s Republic of China; b Institute of High Energy Physics, Chinese Academy of Sciences, Beijing 100049, People’s Republic of China

**Keywords:** covalency, uranyl ion, di­thio­phosphinate, X-ray absorption spectroscopy, density functional theory

## Abstract

The dithiophosphinic acids (HS_2_P*R*_2_) have been used for the selective separation of trivalent actinides (An^III^) from lanthanides (Ln^III^) over the past decades. The substituents on the di­thio­phosphinic acids dramatically impact the separation performance, but the mechanism is still open for debate. In this work, two di­thio­phosphinic acids with significantly different An^III^/Ln^III^ separation performance, *i.e.* di­phenyl di­thio­phosphinic acid (HS_2_PPh_2_) and bis­(*ortho*-trifluoro­methyl­phenyl) di­thio­phosphinic acid [HS_2_P(*o*-CF_3_C_6_H_4_)_2_], are employed to understand the substituent effect on the bonding covalency between the S_2_P*R*_2_^−^ anions (*R* = Ph and *o*-CF_3_C_6_H_4_) and the uranyl ion by sulfur *K*-edge X-ray absorption spectroscopy (XAS) in combination with density functional theory calculations. The two UO_2_(S_2_P*R*_2_)(EtOH) complexes display similar XAS spectra, in which the first pre-edge feature with an intensity of 0.16 is entirely attributed to the transitions from S 1*s* orbitals to the unoccupied molecular orbitals due to the mixing between U 5*f* and S 3*p* orbitals. The Mulliken population analysis indicates that the amount of  S 3*p* character in these orbitals is essentially identical for the UO_2_(S_2_PPh_2_)_2_(EtOH) and UO_2_[S_2_P(*o*-CF_3_C_6_H_4_)_2_]_2_(EtOH) complexes, which is lower than that in the U 6*d*-based orbitals. The essentially identical covalency in U—S bonds for the two UO_2_(S_2_P*R*_2_)_2_(EtOH) complexes are contradictory to the significantly different An^III^/Ln^III^ separation performance of the two di­thio­phosphinic acids, thus the covalency seems to be unable to account for substituent effects in the An^III^/Ln^III^ separation by the di­thio­phosphinic acids. The results in this work provide valuable insight into the understanding of the mechanism in the An^III^/Ln^III^ separation by the di­thio­phosphinic acids.

## Abstract 2

The first pre-edge feature with an intensity of 0.16 in the S *K*-edge X-ray absorption spectra of UO_2_(S_2_P*R*_2_) (*R* = Ph and *o*-CF_3_C_6_H_4_) is entirely attributed to the transitions from S 1*s* orbitals to the unoccupied molecular orbitals due to the mixing between U 5*f* and S 3*p* orbitals.

## 1. Introduction

The selective separation of trivalent actinides (An^III^) and lanthanides (Ln^III^) is one of the most urgent issues for the implementation of the partitioning and transmutation strategy within advanced nuclear fuel cycles, and it is also recognized as a critical challenge in separation science due to the almost identical ionic radii as well as the similar chemical and physical properties between An^III^ and Ln^III^. Soft-donor ligands have demonstrated good performance in An^III^/Ln^III^ separation, in which the di­thio­phosphinic acids (HS_2_P*R*_2_) show great potential in the An^III^/Ln^III^ separation process (Bessen *et al.*, 2020 ▸). For example, bis­(2,4,4-tri­methyl­pentyl)­dithio­phosphinic acid (the purified Cyanex301) in kerosene can extract Am^III^ from Eu^III^ with a separation factor as high as 5000 (Chen *et al.*, 1996 ▸, 2014 ▸). Other di­thio­phosphinic acids with different substituents were also reported for the An^III^/Ln^III^ separation studies (Peterman *et al.*, 2009 ▸, 2010 ▸; Xu *et al.*, 2008 ▸; Wang, Jia, Pan *et al.*, 2013 ▸; Pu *et al.*, 2019 ▸, 2020 ▸; Wang *et al.*, 2013 ▸, 2019 ▸). Strikingly, bis­(*ortho*-tri­fluoro­methyl­phenyl) di­thio­phosphinic acid [HS_2_P(*o*-CF_3_C_6_H_4_)_2_] can afford an Am^III^/Eu^III^ separation factor higher than 10^4^, which is three orders of magnitude higher than that for di­phenyl di­thio­phosphinic acid (HS_2_PPh_2_) (Xu *et al.*, 2008 ▸; Peterman *et al.*, 2010 ▸).

Several factors emerged in the previous studies that are responsible for the substituent effect in the An^III^/Ln^III^ separation by the di­thio­phosphinic acids, including (1) the deprotonation properties of the di­thio­phosphinic acids (Wang *et al.*, 2019 ▸; Pu *et al.*, 2020 ▸), (2) the chemical stoichiometry and structure of the extracted complexes (Pu *et al.*, 2020 ▸; Xu & Rao, 2014 ▸; Greer *et al.*, 2020 ▸; Tian *et al.*, 2002 ▸, 2003 ▸; Weigl *et al.*, 2005 ▸), and (3) the difference in the binding affinity of the ligands to the metals (especially the covalent part) (Keith & Batista, 2012 ▸; Lan *et al.*, 2012 ▸; Cao *et al.*, 2010 ▸; Bhattacharyya *et al.*, 2011 ▸). For instance, the unexpectedly high p*K*_a_ and strong nucleophile for HS_2_P(*o*-CF_3_C_6_H_4_)_2_ may destabilize the anion to a greater extent and increase selectivity towards actinides (Benson *et al.*, 2008 ▸; Leavitt *et al.*, 2008 ▸). Pu *et al.* found that S_2_PPh_2_^−^ formed up to 2:1 complexes with Nd^3+^, whilst S_2_P(*o*-CF_3_C_6_H_4_)_2_^−^ formed up to 3:1 complexes (Pu *et al.*, 2020 ▸). The substituent effect on the bonding covalency between the di­thio­phosphinate ligands and metal ions has been raised as an important factor in driving An^III^/Ln^III^ separation (Keith & Batista, 2012 ▸; Daly, Keith, Batista, Boland, Clark *et al.*, 2012 ▸; Daly, Keith, Batista, Boland, Kozimor *et al.*, 2012 ▸). Daly *et al.* examined the electronic structure of several S_2_P*R*_2_^−^ anions and found that the S_2_P(*o*-CF_3_C_6_H_4_)_2_^−^ anion was a ‘softer’ extractant as compared with the S_2_PPh_2_^−^ anion, which promoted the selectivity towards actinides (Daly, Keith, Batista, Boland, Clark *et al.*, 2012 ▸). Despite the numerous theoretical calculations on modeling the extraction of Ln^III^ and An^III^ by the di­thio­phosphinic acids (Greer *et al.*, 2020 ▸; Lan *et al.*, 2012 ▸; Bhattacharyya *et al.*, 2011 ▸; Diamond *et al.*, 1954 ▸; Choppin, 2002 ▸; Ingram *et al.*, 2008 ▸; Gaunt *et al.*, 2008 ▸; Sadhu & Dolg, 2019 ▸; Kaneko & Watanabe, 2018 ▸; Kaneko *et al.*, 2017 ▸; Cross *et al.*, 2016 ▸; Kaneko *et al.*, 2015 ▸; Jensen & Bond, 2002 ▸; Lehman-Andino *et al.*, 2019 ▸), experimental evaluation on the covalency in *M*—S (where *M* is a metal) chemical bonding in the di­thio­phosphinate complexes, to the best of our knowledge, has not been reported up to now.

In this work, we are motivated to address whether the extent of covalency in the *M*—S bonds would account for the separation performance. The technique of ligand *K*-edge XAS in combination with density functional theory (DFT) calculations is employed. The intensity of the pre-edge features in XAS directly reflects the amount of ligand *p* character in metal-derived molecular orbitals (MOs) and thus the covalency in metal–ligand bonds (Solomon *et al.*, 2005 ▸). This technique has proven to be one of the most versatile and direct spectroscopic techniques to directly probe the mixing of metal *d* and *f* orbitals with ligand *p* orbitals (Kozimor *et al.*, 2008 ▸, 2009 ▸; Minasian *et al.*, 2012 ▸, 2013 ▸, 2014 ▸, 2017 ▸; Spencer *et al.*, 2013 ▸; Löble *et al.*, 2015 ▸; Pemmaraju *et al.*, 2014 ▸; Ha *et al.*, 2017 ▸; Cross *et al.*, 2017 ▸; Donahue *et al.*, 2014 ▸; Su *et al.*, 2018 ▸; Lee *et al.*, 2019 ▸; Smiles *et al.*, 2020 ▸; Sun *et al.*, 2010 ▸; Sarangi *et al.*, 2007 ▸; Queen *et al.*, 2013 ▸). As the direct examination of complexes of the trivalent actinide such as Am^III^ by synchrotron XAS is not feasible due to high radioactivity, in this work we examined the UO_2_(S_2_P*R*_2_)_2_(EtOH) (*R* = Ph, *o*-CF_3_C_6_H_4_) complexes by sulfur *K*-edge XAS to detect the substituent effect on the bonding covalency between the di­thio­phosphinate anions and the uranyl ion. Results in this work will be informative in terms of the substituent effect on the bonding covalency between the di­thio­phosphinate anions and trivalent actinides, and thus helpful for the understanding of the mechanism in the An^III^/Ln^III^ separation by the di­thio­phosphinic acids.

## 2. Results and discussion

### 2.1. Sample preparation

**Figure 1:**
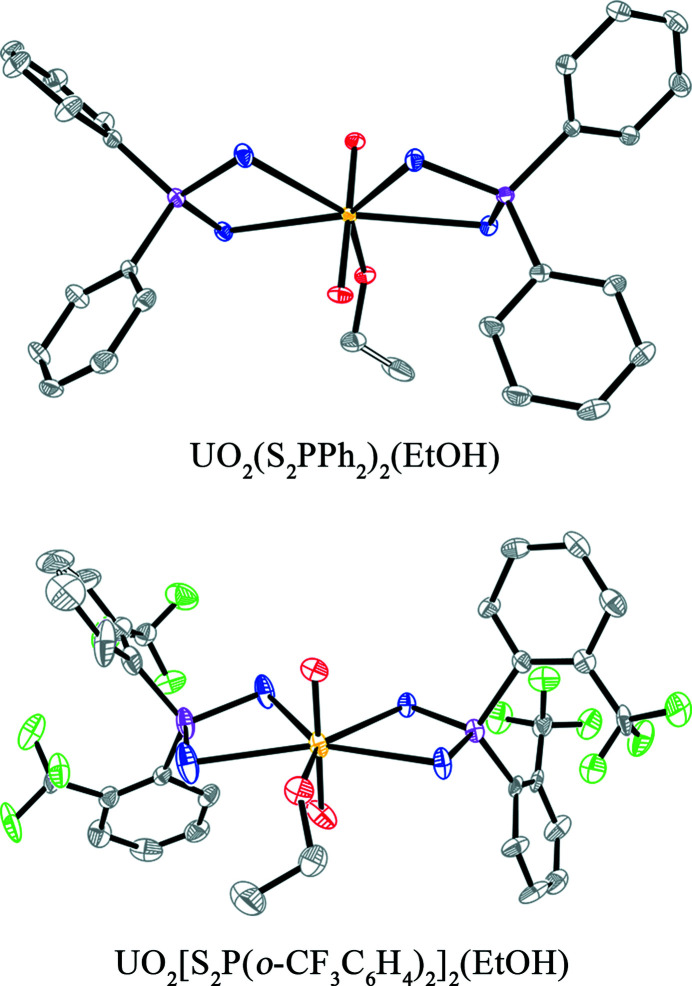
Crystal structure of UO_2_(S_2_P*R*_2_)_2_(EtOH) investigated in this work with thermal ellipsoids drawn at the 30% probability level. H atoms have been omitted for clarity. (U: yellow; S: blue; P: pink; O: red; C: white; F: green.)

Complexes of UO_2_^2+^ with S_2_P*R*_2_^−^ (*R* = Ph, *o*-CF_3_C_6_H_4_) ligands were prepared and isolated as highly pure crystalline solids before the XAS experiments. Note that the complexes of UO_2_^2+^ with the S_2_PPh_2_^−^ ligand have been crystalized previously (Meng *et al.*, 2018 ▸; Pinkerton *et al.*, 1997 ▸; Storey *et al.*, 1983 ▸). However, the crystal structures of the complexes of UO_2_^2+^ with the S_2_P(*o*-CF_3_C_6_H_4_)_2_^−^ ligand have not been reported. In this work, we successfully synthesized single crystals of UO_2_^2+^ with the two S_2_P*R*_2_^−^ ligands. The crystal structures of the UO_2_(S_2_P*R*_2_)_2_(EtOH) complexes are shown in Fig. 1 ▸. Data collection and refinement details are available in Table S1 of the supporting information. Both the two S_2_P*R*_2_^−^ ligands form up to 2:1 complexes with UO_2_^2+^, similar to previous reports (Meng *et al.*, 2018 ▸; Pinkerton *et al.*, 1997 ▸; Storey *et al.*, 1983 ▸). Both the two UO_2_(S_2_P*R*_2_)_2_(EtOH) complexes contain four sulfur atoms from the two bidentate di­thio­phosphinate ligands and one oxygen atom from the coordinated ethanol in the first coordinated sphere of the equatorial plane of UO_2_^2+^. The crystals of the two ligands S_2_P*R*_2_^−^ were also obtained by employing tetra­phenyl­phospho­nium (Ph_4_P^+^) as the cation according to the procedure reported in the literature (Daly, Klaehn *et al.*, 2012 ▸).

The selected bond lengths and bond angles for the crystal structures of the [PPh_4_][S_2_P*R*_2_] ligands and the UO_2_(S_2_P*R*_2_)_2_(EtOH) complexes are provided in Table 1 ▸. The P—S bond lengths are 1.977 and 1.979 Å in S_2_PPh_2_^−^ and S_2_P(*o*-CF_3_C_6_H_4_)_2_^−^, respectively, and these values prolong to 2.010 Å in the two UO_2_(S_2_P*R*_2_)_2_(EtOH) complexes, suggesting comparable bonding interactions of the two S_2_P*R*_2_^−^ ligands with the uranyl ion. The S—U—S bond angles are 70.74° and 70.23° for UO_2_(S_2_Ph_2_)_2_(EtOH) and UO_2_[S_2_P(*o*-CF_3_C_6_H_4_)_2_]_2_(EtOH), respectively, and the S—P—S bond angles are 111.09° and 109.78° for UO_2_(S_2_PPh_2_)_2_(EtOH) and UO_2_[S_2_P(*o*-CF_3_C_6_H_4_)_2_]_2_(EtOH), respectively. The comparable S—U—S and S—P—S bond angles suggest comparable bonding interactions of S_2_PPh_2_^−^ and S_2_P(*o*-CF_3_C_6_H_4_)_2_^−^ with the uranyl ion.

### 2.2. P *K*-edge XAS

**Figure 2:**
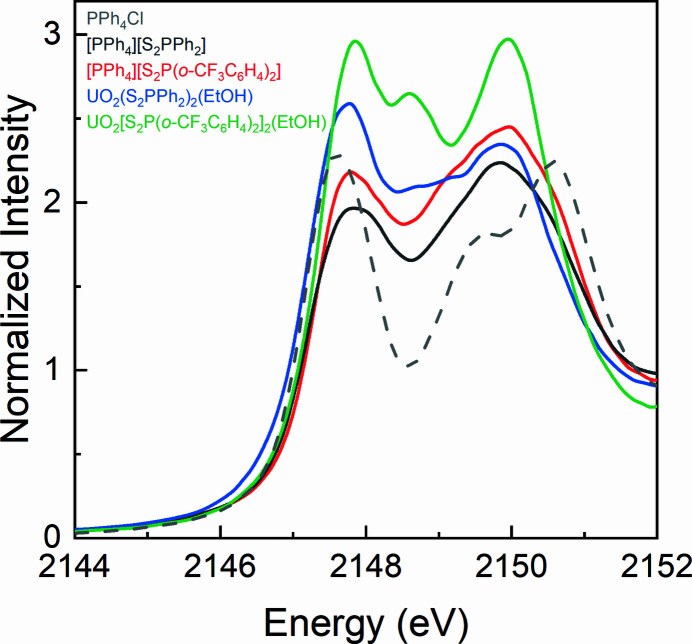
Background-subtracted and normalized P *K*-edge XAS spectra for PPh_4_Cl, [PPh_4_][S_2_P*R*_2_] and UO_2_(S_2_P*R*_2_)_2_(EtOH).

Before the collection of S *K*-edge XAS, we collected the P *K*-edge XAS spectra for the [PPh_4_][S_2_P*R*_2_] ligands and the UO_2_(S_2_P*R*_2_)_2_(EtOH) complexes. The background-subtracted and normalized P *K*-edge XAS spectra are shown in Fig. 2 ▸, and the full spectra are presented in Fig. S1 of the supporting information. The spectrum of PPh_4_Cl was also collected to compare with the spectra of the [PPh_4_][S_2_P*R*_2_] ligands. According to the second derivatives (Fig. S2), the spectrum of PPh_4_Cl contains three pre-edge features at 2147.6, 2149.4 and 2150.7 eV. The spectra of the [PPh_4_][S_2_P*R*_2_] ligands both contain four pre-edge features at about 2147.6, 2149.2 and 2149.8, 2150.5 eV. The spectra of the UO_2_(S_2_P*R*_2_)_2_(EtOH) complexes both contain three pre-edge features at about 2147.6, 2148.9 and 2150 eV. The P *K*-edge XAS spectra are not very informative for the bonding interactions between the uranyl ion and the di­thio­phosphinate, thus collections of the S *K*-edge XAS proceeded for both the [PPh_4_][S_2_P*R*_2_] ligands and the UO_2_(S_2_P*R*_2_)_2_(EtOH) complexes.

### 2.3. S *K*-edge XAS

**Figure 3:**
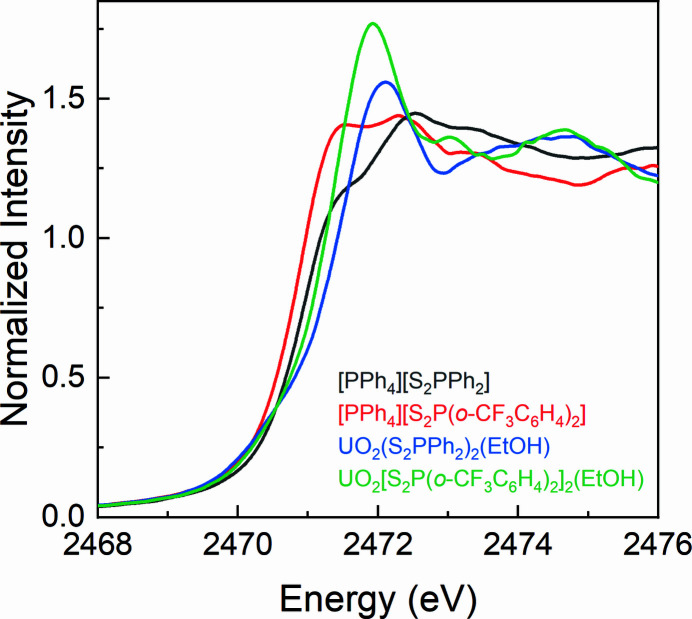
Comparison of the S *K*-edge XAS spectra of [PPh_4_][S_2_P*R*_2_] and UO_2_(S_2_P*R*_2_)_2_(EtOH).

The normalized and background-subtracted S *K*-edge XAS spectra of the [PPh_4_][S_2_P*R*_2_] (*R* = Ph, *o*-CF_3_C_6_H_4_) ligands and the UO_2_(S_2_P*R*_2_)_2_(EtOH) complexes are shown in Fig. 3 ▸. According to the second derivatives (Fig. S4), the spectrum of [PPh_4_][S_2_PPh_2_] contains three pre-edge features at 2471.3, 2472.4 and 2473.6 eV, and the spectrum of [PPh_4_][(S_2_P(*o*-CF_3_C_6_H_4_)_2_] contains four pre-edge features at 2471.3, 2472.4, 2473.4 and 2474.3 eV. This result is in agreement with the previous observations by Daly and co-workers, except that there is no small contribution to the spectra (>2480 eV) from sulfate contaminant (Fig. S3) (Daly, Keith, Batista, Boland, Clark *et al.*, 2012 ▸). The pre-edge features in the spectra of the [PPh_4_][S_2_P*R*_2_] ligands can be assigned to electron transitions from S 1*s* orbitals to the aryl 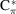 orbitals containing small amounts of sulfur 3*p* character, P—S σ* and P—S σ* + π* orbitals (Daly, Keith, Batista, Boland, Clark *et al.*, 2012 ▸). Compared with the spectra of [PPh_4_][S_2_P*R*_2_], the two UO_2_(S_2_P*R*_2_) (EtOH) complexes display pre-edge features around 2472.0, 2473.5 and 2474.8 eV and also contain a shoulder feature at about 2470.5 eV (Fig. S5). This shoulder feature in the S *K*-edge XAS of the UO_2_(S_2_P*R*_2_)_2_(EtOH) complexes is ascribed to the covalent interaction between the uranyl ion and the S_2_P*R*_2_^−^ ligands.

To quantify the intensities of the pre-edge features, the S *K*-edge XAS spectra of the UO_2_(S_2_P*R*_2_)_2_(EtOH) complexes were modeled using pseudo-Voigt functions with a 1:1 ratio of Lorentzian and Gaussian function contributions and a step function with a 1:1 ratio of arctangent and error function contributions. The energy positions of the features determined by the second derivatives are fixed during the curve-fits (Figs. S5 and S6). The curve-fits parameters are summarized in Table S2.

**Figure 4:**
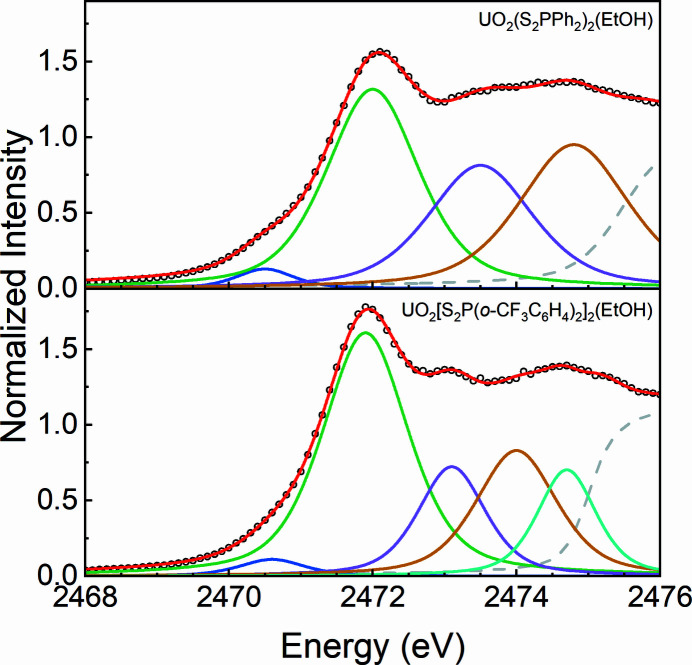
Curve-fitting results of S *K*-edge XAS for UO_2_(S_2_P*R*_2_)_2_(EtOH). The experimental data are shown by black circles, and the total curve fits are shown by red traces. Post-edge residuals (dashed gray traces) are generated by subtracting the pre-edge pseudo-Voigt functions (blue, green, purple, yellow, light blue) from the total curve fits.

The pre-edge region in the spectrum of UO_2_(S_2_PPh_2_)_2_(EtOH) is best modeled by four pseudo-Voigt functions at 2470.5, 2472.0, 2473.5 and 2474.8 eV, and that of UO_2_[S_2_P(*o*-CF_3_C_6_H_4_)_2_]_2_(EtOH) by five pseudo-Voigt functions at 2470.6, 2471.9, 2473.1, 2474.0 and 2474.7 eV, as shown in Fig. 4 ▸ and Table 2 ▸. Note that the shoulder at the energy of 2470.5 eV and 2470.6 eV are both 0.16 of the intensity for UO_2_(S_2_Ph_2_)_2_(EtOH) and UO_2_[S_2_P(*o*-CF_3_C_6_H_4_)_2_)]_2_(EtOH). Resembling the spectra of [PPh_4_][S_2_P*R*_2_], the fourth feature observed in the spectrum of UO_2_(S_2_PPh_2_)_2_(EtOH) at 2474.8 eV splits into two features at 2474.0 and 2474.7 eV in the spectrum of UO_2_[S_2_P(*o*-CF_3_C_6_H_4_)_2_]_2_(EtOH), which may be attributed to the orbital splitting resulting from symmetry change and nonuniform C—P—S angles, according to the results reported by Daly and co-workers (Daly, Keith, Batista, Boland, Clark *et al.*, 2012 ▸).

### 2.4. DFT and time-dependent DFT (TDDFT) calculations

The electronic structure of the S_2_P*R*_2_^−^ anions has been deeply investigated by Daly and co-workers, revealing that the S 3*p* orbitals mix with P 3*p* orbitals to form two σ-type and one π-type P—S bonds in the S_2_P^−^ moiety of S_2_P*R*_2_^−^, and the orbitals of the S_2_P^−^ moiety mix with the σ- and π-type orbitals of the aryl groups (Daly, Keith, Batista, Boland, Clark *et al.*, 2012 ▸; Daly, Keith, Batista, Boland, Kozimor *et al.*, 2012 ▸). Therefore, the S_2_P*R*_2_^−^ ligands can provide both σ and π orbitals to interact with the uranyl ion in the UO_2_(S_2_P*R*_2_)_2_(EtOH) complexes.

**Figure 5:**
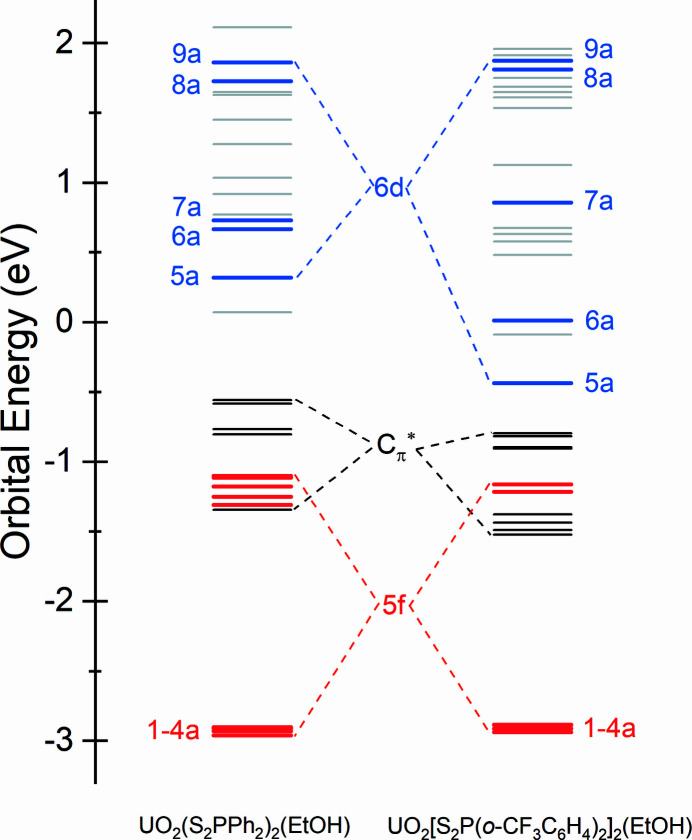
The truncated orbitals energy levels for UO_2_(S_2_P*R*_2_)_2_(EtOH) calculated at the B3LYP/TZ2P level. The red and blue lines denote the orbitals containing primarily U 5*f* and 6*d* character, respectively.

**Figure 6:**
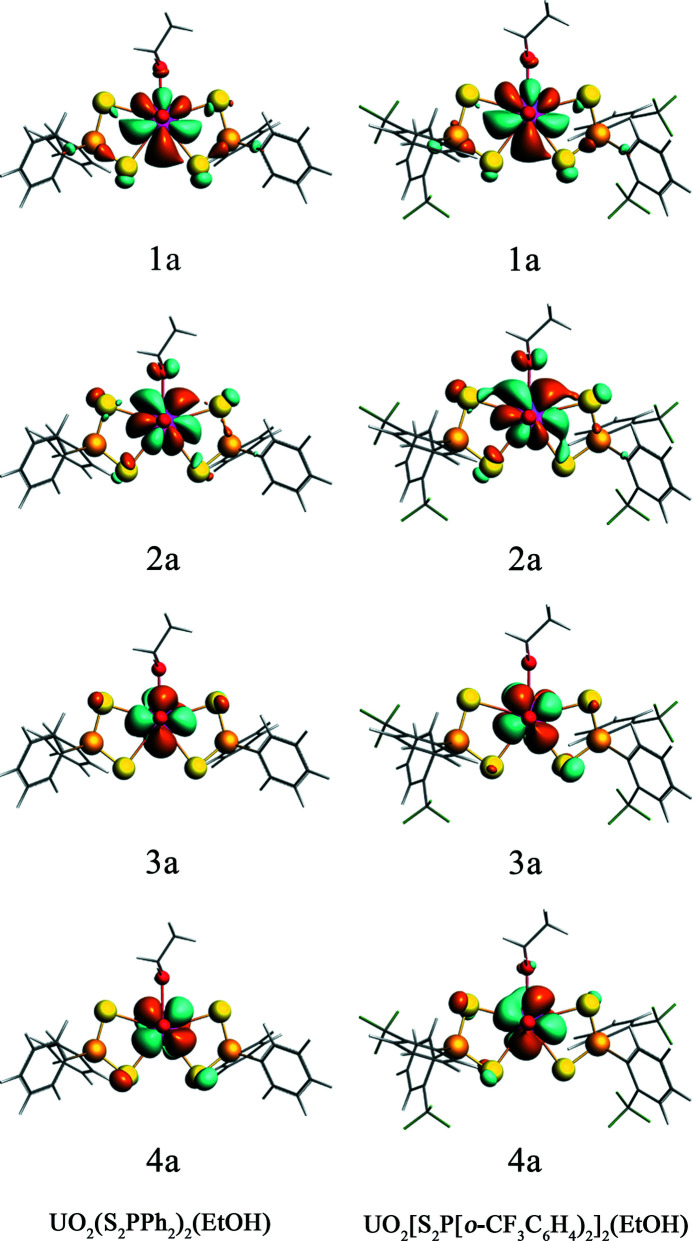
The contours of unoccupied Kohn–Sham orbitals (1–4a) containing primarily U 5*f* character for UO_2_(S_2_P*R*_2_)_2_(EtOH) in 0.02 a.u.

**Figure 7:**
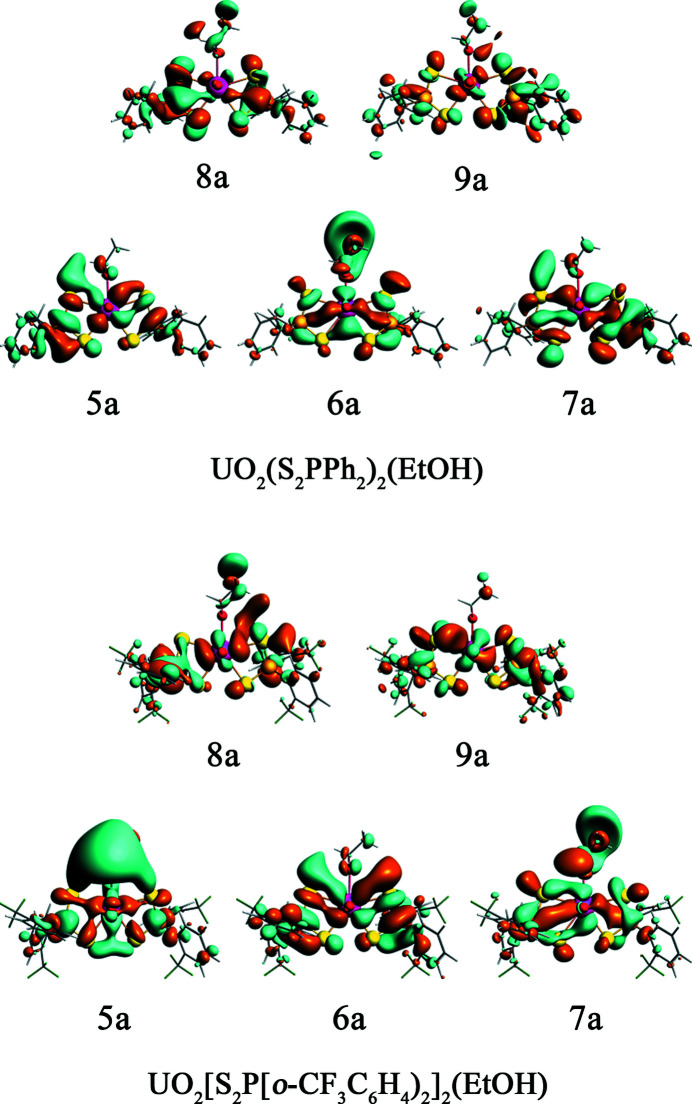
The contours of unoccupied Kohn–Sham orbitals (5–9a) containing primarily U 6*d* character for UO_2_(S_2_P*R*_2_)_2_(EtOH) in 0.02 a.u.

The involvement of both U 5*f* and 6*d* orbitals in the covalent bonds between uranium and axial oxygen (O_yl_) atoms induces a geometrically linear and redox-stable uranyl ion (Denning, 1992 ▸, 2007 ▸; Denning *et al.*, 2002 ▸; Cowie *et al.*, 2019 ▸), around which other ligands are confined to the equatorial plane to interact with the U 5*f* and 6*d* orbitals. DFT calculations were employed to account for the XAS spectra of the UO_2_(S_2_P*R*_2_)_2_(EtOH) complexes in this work. The energy level diagram of the truncated unoccupied MOs for the two UO_2_(S_2_P*R*_2_)_2_(EtOH) complexes is presented in Fig. 5 ▸, and has been shifted by a constant to ensure the energies of the S 1*s* orbitals are equivalent to each other, in order to directly compare with the S *K*-edge XAS. The U 5*f*- and 6*d*-dominant MOs are shown by red and blue lines, respectively. There are four orbitals (1–4a) belonging to the σ- and π-type mixing of the S_2_P orbitals with U 5*f* orbitals near −3 eV, and the contours of these four orbitals are illustrated in Fig. 6 ▸. Other U 5*f*-dominant MOs locate around −1.25 eV showing the orbital mixing between U 5*f* and S 3*p* orbitals, blending in with some orbitals (black lines, ranging from −1.5 eV to −0.5 eV) that contain significant phenyl character ( 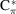 ) and only small contributions from S_2_P fragment orbitals. The U 6*d*-dominant MOs (5–9a, blue lines) are distributed ranging from −0.5 eV to 2.0 eV, interspersing with some σ* S_2_P orbitals (gray lines) containing little S 3*p* character. The contours of the U 6*d*-dominant MOs (5–9a) for UO_2_(S_2_P*R*_2_)_2_(EtOH) are illustrated in Fig. 7 ▸, and those of the orbitals containing significant phenyl character ( 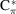 ) for UO_2_(S_2_P*R*_2_)_2_(EtOH) are illustrated in Fig. S7.

According to the orbital energies obtained by the ground-state DFT calculations in Fig. 5 ▸, the first pre-edge features around 2470.5 eV in the S *K*-edge XAS of the UO_2_(S_2_P*R*_2_)_2_(EtOH) complexes in Fig. 4 ▸ are reasonably assigned to the transitions from S 1*s* orbitals to the U 5*f*-dominant MOs (1–4a). The second pre-edge features around 2472.0 eV are dominated by the transitions associated with primarily phenyl character ( 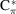 ). The other pre-edge features at energy from 2473 to 2475 eV are contributed by U 6*d*-dominant MOs and orbitals containing little S 3*p* components without U 5*f* or 6*d* character.

**Figure 8:**
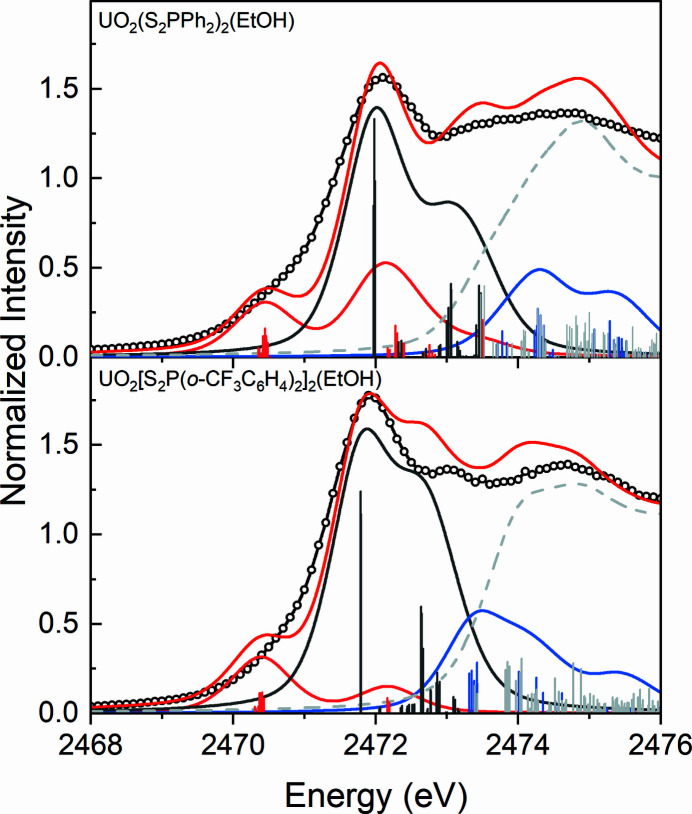
Comparison of the simulated spectra obtained by calculations (red) with the experimental S *K*-edge XAS data for UO_2_(S_2_P*R*_2_)_2_(EtOH) (black). The purple, blue, orange and light gray bars represent the energies and oscillator strengths for the calculated transitions involving U 5*f*, 6*d*, 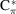 and S_2_P orbitals containing little S 3*p* character without U 5*f* or 6*d* character, respectively.

The S *K*-edge XAS spectra for the UO_2_(S_2_P*R*_2_)_2_(EtOH) complexes were simulated by TDDFT calculations to directly compare with the experiment XAS (Fig. 8 ▸). The simulated spectra for both UO_2_(S_2_P*R*_2_)_2_(EtOH) complexes have been shifted by +49.7 eV to account for the omission of the atomic and extra-atomic relaxation associated with the core excitation, relativistic stabilization, and errors associated with the functional (Martin & Shirley, 1977 ▸; Segala & Chong, 2010 ▸).

The simulated spectra are in good agreement with the experimental spectra. The transitions around 2470.5 eV in the simulated spectra are observed for both the UO_2_(S_2_P*R*_2_)_2_(EtOH) complexes, which is entirely attributed to the transitions from S 1*s* orbitals to the U 5*f*-dominant unoccupied MOs (1–4a), indicating the covalent mixing between S 3*p* orbitals and U 5*f* orbitals. The transitions from S 1*s* orbitals to the primarily phenyl character ( 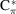 ) unoccupied MOs and little U 5*f* character unoccupied MOs both contribute to the pre-edge features around 2472.0 eV obtained by curve-fits in Fig. 4 ▸. The other pre-edge features above 2473 eV are associated with the transitions to the U 6*d*-dominant MOs and to the orbitals containing little S 3*p* components without U 5*f* or 6*d* character.

### 2.5. Evaluation of the bonding covalency between the uranyl ion and the S_2_P*R*_2_^−^ ligands

It has been well known that the amount of ligand *n* *p* character in metal-derived MOs can be determined from the intensities observed in the pre-edge features in the ligand *K*-edge XAS (Solomon *et al.*, 2005 ▸; Barton *et al.*, 2015 ▸). Generally, an intensity standard is used to convert the experimental intensity of a pre-edge feature to the amount of ligand *n* *p* character in metal–ligand bonds. For example, an intensity of 0.53 = 7.5% Cl 3*p*-character per bond obtained from Cs_2_CuCl_4_ is used as the Cl *K*-edge XAS intensity standard (Solomon *et al.*, 2005 ▸). The standards for thiol­ate (S*R* ^−^) (Shadle *et al.*, 1993 ▸), sulfide (S^2−^) (Rose *et al.*, 1999 ▸) and enedi­thiol­ate (S_2_ *R*_2_^2−^) (Szilagyi *et al.*, 2003 ▸) have been established to evaluate the amount of S 3*p* character in *M*—S bonds. Since the intrinsic transition dipole for the S 1*s* → 3*p* excitation is dependent on the effective nuclear charge *Z*_eff_ (S) for each S-ligand (Solomon *et al.*, 2005 ▸), it is not appropriate to directly use the standard for thiol­ate, sulfide or enedi­thiol­ate to convert the intensities in this work (S in the form of S_2_P*R*_2_^−^) to % S 3*p* character. Therefore, we herein use the data from Mulliken population analysis that are associated with the experimental XAS data to evaluate the bonding covalency between the uranyl ion and the S_2_P*R*_2_^−^ ligands (Table 3 ▸).

The DFT calculations show that the amount of S 3*p* character in the U 5*f*-dominant unoccupied MOs of 1a, 2a, 3a and 4a for UO_2_(S_2_PPh)_2_(EtOH) is 2.68%, 3.75%, 1.95% and 3.04%, respectively. For UO_2_{[S_2_P(*o*-CF_3_C_6_H_4_)]_2_}_2_(EtOH), these values are 1.95%, 4.09%, 2.48% and 2.73%, respectively. The total amount of S 3*p* character of the orbitals of 1a, 2a, 3a and 4a are 11.42% and 11.25%, corresponding to 2.86% and 2.81% per U—S bond in UO_2_(S_2_PPh)_2_(EtOH) and UO_2_{[S_2_P(*o*-CF_3_C_6_H_4_)]_2_}_2_(EtOH), respectively. The DFT calculations also show that the amount of S 3*p* character in the U 6*d*-dominant unoccupied MOs of 5a, 6a, 7a, 8a and 9a for UO_2_(S_2_PPh)_2_(EtOH) is 12.11%, 9.0%, 13.43%, 14.43% and 16.78%, respectively. For UO_2_[(S_2_P(*o*-CF_3_C_6_H_4_))_2_]_2_(EtOH), these values are 13.00%, 21.93%, 8.94%, 6.29% and 13.39%, respectively. The average amount of S 3*p* character in U 6*d*-based orbitals (5–9a) obtained from the DFT calculations are 13.15% and 12.71% for UO_2_(S_2_PPh)_2_(EtOH) and UO_2_{[S_2_P(*o*-CF_3_C_6_H_4_)]_2_}_2_(EtOH), respectively, thus the S 3*p* orbitals are engaged more in the U 6*d* orbitals than that in the U 5*f* orbitals, consistent with the previous reports that the 6*d* orbitals play a significant role in actinide bonding relative to the 5*f* orbitals (Minasian *et al.*, 2012 ▸; Pepper & Bursten, 1991 ▸; Su *et al.*, 2018 ▸; Cross *et al.*, 2017 ▸). The average amount of S 3*p* character in the orbitals with primarily phenyl character ( 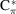 ) obtained from the DFT calculations is 2.25% and 3.65% for UO_2_(S_2_PPh)_2_(EtOH) and UO_2_{[S_2_P(*o*-CF_3_C_6_H_4_)]_2_}_2_(EtOH), respectively, indicating an important contribution to the pre-edge features. Although only the first pre-edge feature around 2470.5 eV in each spectrum is exclusively attributed to the transitions from S 1*s* orbitals to the U 5*f*-dominant unoccupied MOs (1–4a), the XAS data and DFT calculations both suggest that the mixing of U 5*f* and 6*d* orbitals with S 3*p* orbitals are similar in the two UO_2_(S_2_P*R*_2_)_2_(EtOH) complexes, indicating that introduction of *o*-CF_3_ into phenyl has little effect on the covalent bonding between S_2_P*R*_2_^−^ and UO_2_^2+^.

## 3. Conclusion

A combination of the S *K*-edge XAS technique and DFT calculations has been conducted on UO_2_(S_2_P*R*_2_)_2_(EtOH) (*R* = Ph and *o*-CF_3_C_6_H_4_) complexes to obtain direct insight into the contributions of U 6*d* and especially 5*f* orbitals to the covalency in the U—S bonds, in order to illuminate the role of the bonding covalency in the An^III^/Ln^III^ separation by the di­thio­phosphinic acids. The two UO_2_(S_2_P*R*_2_)_2_(EtOH) complexes display similar pre-edge features in the S *K*-edge XAS, the first of which is entirely attributed to the transitions from S 1*s* orbitals to the U 5*f* orbitals mixing with the S 3*p* orbitals. Curve-fitting analysis indicates identical intensities of 0.16 for the first pre-edge feature of the two UO_2_(S_2_P*R*_2_)_2_(EtOH) complexes. Consistently, the amounts of S 3*p* character per U—S bond for UO_2_(S_2_Ph_2_)_2_(EtOH) and UO_2_[S_2_P(*o*-CF_3_C_6_H_4_)_2_]_2_(EtOH) by Mulliken population analysis are essentially identical to each other. In addition, the DFT calculations show that the amounts of S 3*p* character in U 6*d*-based orbitals are also nearly equivalent for the two UO_2_(S_2_P*R*_2_)_2_(EtOH) complexes. The XAS data and DFT calculations demonstrate essentially identical bonding covalency in the two UO_2_(S_2_P*R*_2_)_2_(EtOH) complexes, indicating that the introduction of *o*-CF_3_ into phenyl has little effect on the covalent bonding between the S_2_P*R*_2_^−^ ligands and UO_2_^2+^. The essentially identical covalency in the U—S bonds for the two UO_2_(S_2_P*R*_2_)_2_(EtOH) complexes are contradictory to the significantly different An^III^/Ln^III^ separation performance of the two di­thio­phosphinic acids. The *M*—S bonding covalency seems to be unable to account for the substituent effect in the An^III^/Ln^III^ separation by the di­thio­phosphinic acids. According to the results in the previous work as mentioned in the *Introduction* [Sec sec1], we speculate that the different chemical stoichiometry and structure of the extracted complexes should be the main reason for the significantly different separation performance of HS_2_PPh_2_ and HS_2_P(*o*-CF_3_C_6_H_4_)_2_ in the An^III^/Ln^III^ separation. Nevertheless, it is worthwhile conducting an experimental investigation on the bonding covalency between the trivalent actinides and di­thio­phosphinate ligands with different substituents in the future.

## 4. Experimental section

### 4.1. Synthesis of UO_2_(S_2_P*R*_2_)_2_(EtOH)

All manipulations were carried out in a glove box under an atmosphere of nitro­gen to rigorously exclude air and moisture. Ethanol was dried and degassed by the solvent purification system, and transferred to the glove box without exposure to air. Super dry di­chloro­methane was stored over activated molecular sieves prior to use. Single crystals of [PPh_4_][S_2_P*R*_2_] suitable for X-ray diffraction characterization were obtained by recrystallization from a 1:1 aceto­nitrile/toluene solution under air and at ambient conditions, according to the procedure reported in the literature (Daly, Klaehn *et al.*, 2012 ▸). In synthesizing the single crystals of the two UO_2_(S_2_P*R*_2_)_2_(EtOH) complexes, two di­thio­phosphinate ligands in the ammonium form [NH_4_][S_2_P*R*_2_] were used according to the previous procedures (Daly, Klaehn *et al.*, 2012 ▸).

**UO_2_(S_2_PPh_2_)_2_(EtOH)**. Single crystals of the UO_2_(S_2_P*R*_2_)_2_(EtOH) complexes were prepared using the reported procedures with slight modifications (Meng *et al.*, 2018 ▸; Pinkerton *et al.*, 1997 ▸; Storey *et al.*, 1983 ▸). A solution of [NH_4_][S_2_PPh_2_] (54.2 mg, 0.203 mmol) in ethanol (2 ml) was mixed with a solution of UO_2_Cl_2_ (35.8 mg) in ethanol (2 ml). The mixture was stirred for 20 min at 70°C to give an orange solution. A slight white precipitate was generated over the course of the reaction. The orange solution was taken to dryness and the resulting complexes were extracted from the white–orange solid residue with di­chloro­methane (10 ml). The products were recrystallized after solvent removal from 1.5 ml ethanol. After one week, single crystals suitable for X-ray diffraction were obtained at room temperature. IR (cm^−1^, solid sample on ATR cell): 923 (asymmetric O=U=O stretching), 559 (symmetric PS_2_ stretching), 631 (asymmetric PS_2_ stretching). Raman (cm^−1^): 840 (symmetric O=U=O stretching). Anal. Calcd for C_26_H_26_O_3_P_2_S_4_U: C, 38.33; H, 3.22. Found: C, 38.24; H, 3.27.

**[UO_2_[S_2_P(*o*-CF_3_C_6_H_4_)_2_]_2_(EtOH)]·EtOH**. Single crystals of UO_2_[S_2_P(*o*-CF_3_C_6_H_4_)_2_]_2_(EtOH) were prepared as described above for UO_2_(S_2_PPh_2_)_2_(EtOH) from [NH_4_][S_2_P(*o*-CF_3_C_6_H_4_)_2_] (71.4 mg, 0.177 mmol) and UO_2_Cl_2_ (35.4 mg). IR (cm^−1^, solid sample on ATR cell): 930 (asymmetric O=U=O stretching), 560 (symmetric PS_2_ stretching), 646 (asymmetric PS_2_ stretching). Raman (cm^−1^): 845 (symmetric O=U=O stretching). Anal. Calcd for C_32_H_28_F_12_O_4_P_2_S_4_U: C, 33.93; H, 2.49. Found: C, 33.79; H, 2.66.

### 4.2. X-ray crystallography

The single-crystal X-ray diffraction data for [PPh_4_][S_2_P*R*_2_] and UO_2_(S_2_P*R*_2_)_2_(EtOH) complexes were collected on a Rigaku Super Nova, Dual, Cu at zero, AtlasS2 diffractometer. The measurements were performed with Cu/*K*α (λ = 1.54184 Å) or Mo/*K*α (λ = 0.71073 Å) radiation. All crystals were kept at 173 K during data collection. Data collection and reduction were carried out in *CrysAlisPro*, Version 1.171.39.46 (Rigaku Oxford Diffraction, 2018 ▸). A multi-scan method for absorption corrections was applied to the data sets. All the structures were solved by intrinsic phasing method and refined by full matrix least-squares techniques with anisotropic temperature factors of all non-hydrogen atoms on *F* ^2^, using the *SHELX-97* and *Olex2-1.2* program (Sheldrick, 2008 ▸; Dolomanov *et al.*, 2009 ▸). All H atoms were refined with anisotropic displacement parameters. The H atoms were placed in ideal sites and were not refined for good refinement convergence. Further data collection and refinement details are summarized in Table S1. The CIF files containing the supplementary crystallographic data for [PPh_4_][S_2_P*R*_2_] and UO_2_(S_2_P*R*_2_)_2_(EtOH) are available through the Cambridge Crystallographic Data Centre (CCDC 2103608–2103611).

### 4.3. XAS measurements and data analysis

The S and P *K*-edge XAS measurements were conducted on beamline 4B7A of Beijing Synchrotron Radiation Facility (BSRF) over an energy range from 1750 eV to 6000 eV. The energy of the electron beam is 2.5 GeV in the storage ring with a maximum beam current of 250 mA. A beam spot tightly focused at a sample is about 1.5 mm × 0.4 mm, and the measured flux is over 3 × 10^10^ photons s^−1^ (250 mA)^−1^ (Zheng *et al.*, 2014 ▸). The samples were measured by partial fluorescence yield mode using a 13-element Si (Li) array detector.

For S and P *K*-edge XAS measurements, single-crystal samples were finely ground into a homogeneous powder which was dispersed as thinly as possible on the carbon tape. The energy scale in the S and P *K*-edge XAS was calibrated by using Na_2_S_2_O_3_ and Na_4_P_2_O_7_ standards, respectively, which was repeatedly analyzed at intervals between sample scans. All spectra were collected in duplicate at least twice to obtain adequate statistics. Spectra showed no signs of radiation damage and were reproduced over multiple regions of the sample.

Background subtraction and normalization of S and P *K*-edge XAS data were manipulated using the *Athena* interface in the *Demeter* software program (Ravel & Newville, 2005 ▸). In a typical example, a line was fit to the pre-edge region and then subtracted from the experimental data to eliminate the background of the spectrum. The data were normalized to a unit step height by fitting a second-order polynomial to the post-edge region of the spectrum. Curve-fitting of the S *K*-edge XAS was performed using the program *IGOR Pro 8.04* and a modified version of *EDG_FIT* (George, 2001 ▸). Second-derivative spectra were used as guides to determine the number and position of peaks. Pre-edge and rising edge features were modeled by symmetrically constrained pseudo-Voigt line shapes with a fixed 1:1 Lorentzian to Gaussian ratio and a step function with a 1:1 ratio of arctangent and error function, respectively. Fits were performed over several energy ranges. The quality of each curve-fit was determined by evaluating changes in χ^2^ and by inspecting the residual intensity, which is obtained by subtracting the fit from the experiment data and should resemble a horizontal line at zero. The area under the pre-edge features (defined as the intensity) was used as the transition intensity.

### 4.4. DFT calculations

All DFT calculations were carried out with the *Amsterdam Density Functional* (*ADF 2019*) program (Baerends *et al.*, 2019 ▸; te Velde *et al.*, 2001 ▸), employing the B3LYP hybrid functional (Becke, 1988 ▸; Lee *et al.*, 1988 ▸). The all-electron Slater-type orbital (STO) basis sets of triple-ζ augmented by two sets of polarization functions (TZ2P) were adapted for the description of all atoms. The zero-order regular approximation (ZORA) approach was used to account for the scalar relativistic (SR) effects (Faas *et al.*, 1995 ▸). Mulliken population analyses were conducted on particular MOs to obtain the reported orbital populations in the two UO_2_(S_2_P*R*_2_)_2_(EtOH) complexes (Mulliken, 1955 ▸).

The S *K*-edge XAS spectra for all complexes were simulated by TDDFT using the Davidson method. The simulated spectra were obtained by calculating core electron excitations originating from S 1*s* dominated MOs to virtual MOs at the optimized crystal structure. Only excitations from S 1*s* core levels to virtual orbitals were analyzed by restricting the energy range of core level and virtual orbitals involved in excitation. The calculated oscillator strengths were evenly broadened with a pseudo-Voigt functions with a 1:1 ratio of Lorentzian and Gaussian function contributions of 1 eV full width at half-maximum to generate the simulated absorption spectra. An energy shift of +49.7 eV was applied for the simulated spectra to account for the omission of atomic and extra-atomic relaxation associated with the core excitation, relativistic stabilization, and errors associated with the functional, according to the literature (Martin & Shirley, 1977 ▸; Segala & Chong, 2010 ▸).

## Supplementary Material

Crystal structure: contains datablock(s) I, II, III, IV. DOI: 10.1107/S160057752101198X/yw5001sup1.cif

Structure factors: contains datablock(s) I. DOI: 10.1107/S160057752101198X/yw5001Isup2.hkl

Structure factors: contains datablock(s) II. DOI: 10.1107/S160057752101198X/yw5001IIsup3.hkl

Structure factors: contains datablock(s) III. DOI: 10.1107/S160057752101198X/yw5001IIIsup4.hkl

Structure factors: contains datablock(s) IV. DOI: 10.1107/S160057752101198X/yw5001IVsup5.hkl

Supporting information: Tables S1 and S2; Figures S1 to S7. DOI: 10.1107/S160057752101198X/yw5001sup6.pdf

CCDC references: 2103608, 2103609, 2103610, 2103611

## Figures and Tables

**Table 1:** Selected bond lengths (Å) and bond angles (°) for [PPh_4_][S_2_P*R*_2_] and UO_2_(S_2_P*R*_2_)_2_(EtOH) in the crystal structures

	Bond length			Bond angle		
Compound	U−O_yl_	U—S	P—S	O−U−O	S—U—S	S—P—S
[PPh_4_][S_2_PPh_2_]	–	–	1.977 (1)	–	–	117.80 (4)
[PPh_4_][S_2_P(*o*-CF_3_C_6_H_4_)_2_]	–	–	1.979 (2)	–	–	116.79 (3)
UO_2_(S_2_PPh_2_)_2_(EtOH)	1.765 (1)	2.863 (29)	2.010 (26)	175.77 (10)	70.74 (2)	111.09 (5)
UO_2_[S_2_P(*o*-CF_3_C_6_H_4_)_2_]_2_(EtOH)	1.740 (42)	2.858 (36)	2.010 (11)	177.1 (10)	70.23 (19)	109.78 (93)

**Table 2:** The energy and intensity obtained by curve-fitting of the S *K*-edge XAS for UO_2_(S_2_P*R*_2_)_2_

Compound	Energy (eV)	Intensity
UO_2_(S_2_PPh_2_)_2_(EtOH)	2470.5	0.16
	2472.0	2.65
	2473.5	1.85
	2474.8	2.26
UO_2_[S_2_P(*o*-CF_3_C_6_H_4_)_2_]_2_(EtOH)	2470.6	0.16
	2471.9	2.99
	2473.1	1.05
	2474.0	1.46
	2474.7	0.89

**Table 3:** Mulliken population analysis for unoccupied MOs of the UO_2_(S_2_P*R*_2_)_2_(EtOH)

Compound	MO		Energy (eV)	% S 3*p*	% S 3*p* average
UO_2_(S_2_PPh_2_)_2_(EtOH)	5*f*	1a	−2.96	2.68	2.86
		2a	−2.93	3.75	
		3a	−2.91	1.95	
		4a	−2.90	3.04	

	6*d*	5a	0.32	12.11	13.15
		6a	0.67	9.00	
		7a	0.73	13.43	
		8a	1.73	14.43	
		9a	1.86	16.78	

	{\rm{C}}_\pi^{\,\ast}	10a	−1.34	9.25	2.25
		11a	−0.80	0.39	
		12a	−0.77	0.62	
		13a	−0.59	0.51	
		14a	−0.56	0.50	

UO_2_[S_2_P(*o*-CF_3_C_6_H_4_)_2_]_2_(EtOH)	5*f*	1a	−3.30	1.95	2.81
		2a	−3.27	4.09	
		3a	−3.25	2.48	
		4a	−3.24	2.73	

	6*d*	5a	−0.80	13.00	12.71
		6a	−0.35	21.93	
		7a	0.50	8.94	
		8a	1.45	6.29	
		9a	1.51	13.39	

	{\rm{C}}_\pi^{\,\ast}	10a	−1.88	6.95	3.65
		11a	−1.85	4.78	
		12a	−1.80	6.15	
		13a	−1.74	5.48	
		14a	−1.27	0.73	
		15a	−1.26	0.80	
		16a	−1.18	2.77	
		17a	−1.16	1.54	
