# Supplementary material for: Covalency between the uranyl ion and di­thio­phosphinate by sulfur K-edge X-ray absorption spectroscopy and density functional theory
Source: J Synchrotron Radiat. 2022 Jan 1;29(Pt 1):11–20. doi: 10.1107/S160057752101198X (PMC8733989; doi:10.1107/S160057752101198X)
Supplement: Supplementary file 6 [file s-29-00011-sup6.pdf]

JOURNAL OF SYNCHROTRON RADIATION

Volume 29 (2022)

Supporting information for article:

Covalency between the Uranyl Ion and Dithiophosphinate by Sulfur K-edge X-ray Absorption Spectroscopy and Density Functional Theory

Yusheng Zhang, Wuhua Duan, Qiang Wang, Lei Zheng, Jianchen Wang, Jing Chen and Taoxiang Sun

Table S1 Crystallographic data for $[PPh_4][S_2PR_2]$ and $UO_2(S_2PR_2)_2(EtOH)$.

	$[PPh_4][S_2PPh_2]$	$[PPh_4][S_2P(o\text{-}CF_3C_6H_4)_2]$	$UO_2(S_2PPh_2)_2(EtOH)$	$UO_2[S_2P(o\text{-}CF_3C_6H_4)_2]_2(EtOH)\cdot EtOH$
Chemical formula	$C_{36}H_{30}P_2S_2$	$C_{38}H_{28}F_6P_2S_2$	$C_{26}H_{26}O_3P_2S_4U$	$C_{32}H_{28}F_{12}O_4P_2S_4U$
Formula weight	588.66	724.66	814.68	1132.75
T (K)	172.99(10)	172.99(10)	173.00(10)	173.00(10)
Crystal system	monoclinic	triclinic	triclinic	orthorhombic
Space group	$P2_1/n$	P-1	P-1	$Pca2_1$
a (Å)	14.5406(12)	8.7782(3)	11.5443(3)	16.5607(6)
b (Å)	13.2405(7)	13.2640(5)	11.6402(3)	8.1303(3)
c (Å)	17.1497(12)	15.3166(4)	13.0973(3)	59.7044(13)
α (°)	90	87.778(3)	113.227(2)	90
β (°)	108.962(8)	88.384(3)	110.729(2)	90
γ (°)	90	75.173(3)	92.865(2)	90
V (Å^3)	3122.6(4)	1722.39(10)	1475.39(7)	8038.8(5)
Z	4	2	2	8
ρ_{calcd} (g/cm^3)	1.252	1.397	1.834	1.872
Crystal color	colorless	colorless	clear orange	clear yellow
Crystal description	plate	needle	plate	plate
Crystal size (mm^3)	0.2 × 0.15 × 0.02	0.3 × 0.15 × 0.1	0.35 × 0.3 × 0.05	0.16 × 0.1 × 0.02
λ (Å)	0.71073	1.54184	0.71073	1.54184
2θ range (°)	6.42 – 60.51	6.898 – 149.77	6.682 – 60.878	8.886 – 143.14
Index ranges	$-20 \leq h \leq 18$	$-10 \leq h \leq 10$	$-15 \leq h \leq 16$	$-20 \leq h \leq 9$
	$-13 \leq k \leq 18$	$-16 \leq k \leq 16$	$-16 \leq k \leq 16$	$-9 \leq k \leq 7$

	−24 ≤ l ≤ 20	−12 ≤ l ≤ 19	−18 ≤ l ≤ 18	−68 ≤ l ≤ 72
Reflections collected	23257	12466	27638	19109
Independent reflections	8252	6868	8094	11827
Unique reflections (R_{int})	0.0639	0.0333	0.0467	0.0607
Data/restraints/parameters	8252/12/361	6868/0/433	8094/3/340	11827/238/1018
R_1, wR_2 ($I \geq 2\sigma(I)$)	0.0669, 0.1122	0.0410, 0.1049	0.0310, 0.0573	0.0672, 0.1675
R_1, ωR_2 (all data)	0.1177, 0.1312	0.0485, 0.1114	0.0375, 0.0598	0.0791, 0.1779
Goodness-of-fit on F^2	1.044	1.0342	1.043	1.036

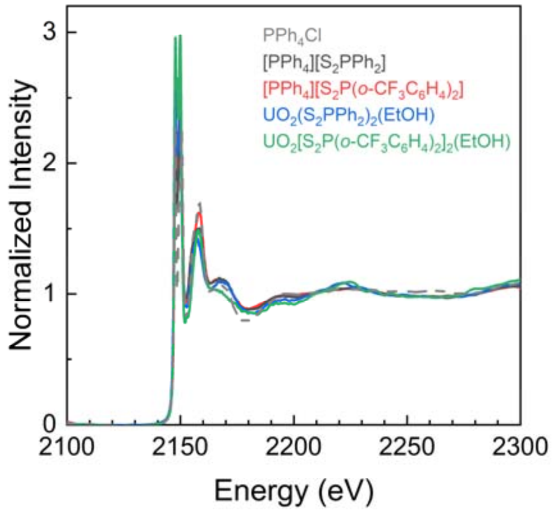

Figure S1 P K-edge XAS spectra of PPh_4Cl, $[PPh_4][S_2PR_2]$, and $UO_2(S_2PR_2)_2(EtOH)$.

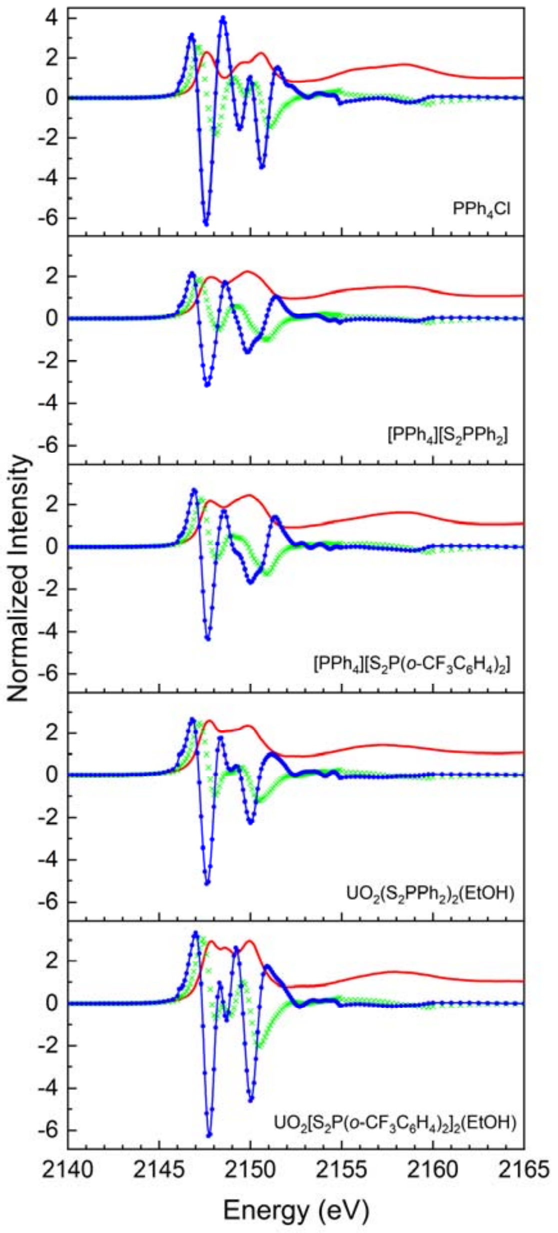

Figure S2 First (green) and second (blue) derivatives of the P K-edge XAS spectra of PPh_4Cl, $[PPh_4][S_2PR_2]$, and $UO_2(S_2PR_2)_2(EtOH)$.

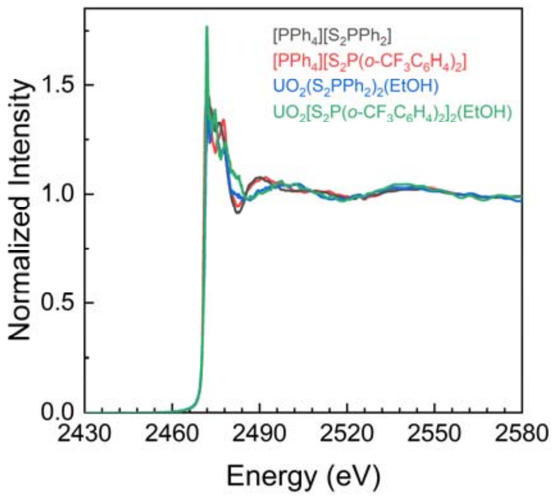

Figure S3 S K-edge XAS spectra of $[PPh_4][S_2PR_2]$ and $UO_2(S_2PR_2)_2(EtOH)$.

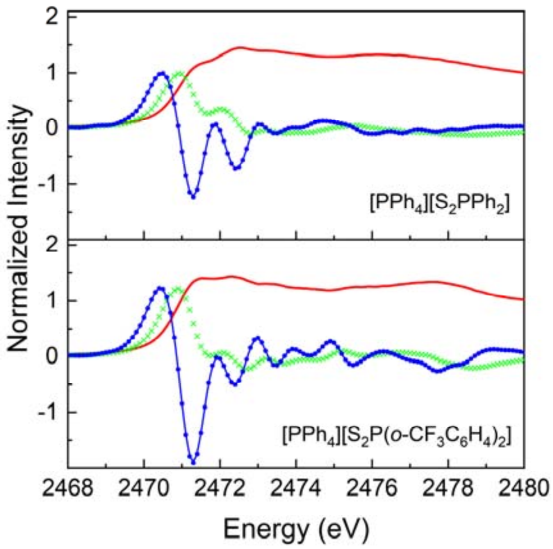

Figure S4 First (green) and second (blue) derivatives of the S K-edge XAS spectra of $[PPh_4][S_2PR_2]$.

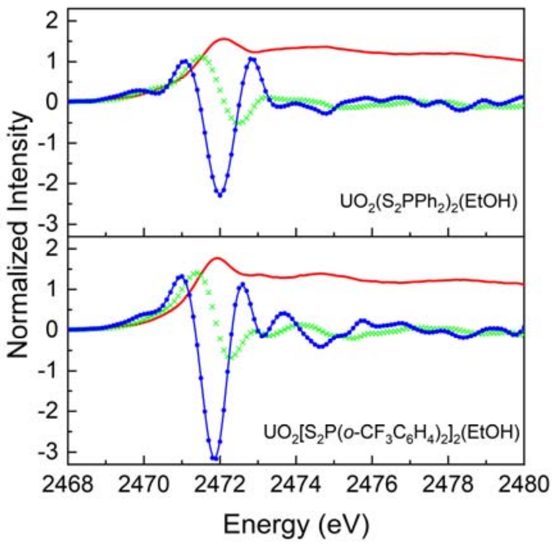

Figure S5 First (green) and second (blue) derivatives of the S K-edge XAS spectra of $UO_2(S_2PR_2)_2(EtOH)$.

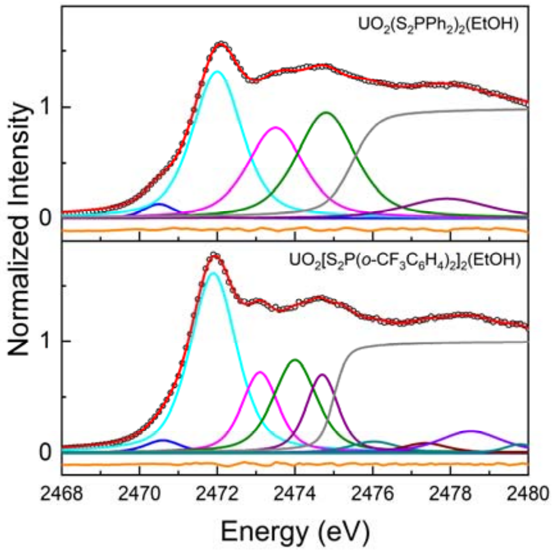

Figure S6 Curve-fitting results for the S K-edge XAS for $UO_2(S_2PR_2)_2(EtOH)$. The spectra were modeled using pseudo-Voigt line shapes (blue, cyan, magenta, olive, purple, dark cyan, wine and violet traces) with a fixed 1:1 Lorentzian to Gaussian ratio and a step function (gray traces) with a 1:1 ratio of arctangent and error function contributions. The experimental data are shown in black circles, and the total curve fits are shown in red traces. Total residual data (orange, offset) was determined by subtracting the curve fit from the experimental data.

Table S2 Curve-fitting parameters for the S K-edge XAS of the $UO_2(S_2PR_2)_2(EtOH)$ complexes. Only the pre-edge features shown in Fig. S6 are listed.

Compound	$UO_2(S_2PPh_2)_2(EtOH)$	$UO_2[S_2P(o\text{-}CF_3C_6H_4)_2]_2(EtOH)$
Step function amplitude	0.5	0.5
Step function energy (eV)	2475.5 ± 0.0148	2475.0 ± 0.00761
Step function half-width	0.55159 ± 0.027	0.28762 ± 0.019
Peak 1 Amplitude	0.13138 ± 0.00592	0.11242 ± 0.00554
Peak 1 Energy (eV)	2470.5	2470.6
Peak 1 Half-Width (eV)	0.47269 ± 0.0306	0.53499 ± 0.0303
Peak 2 Amplitude	1.3233 ± 0.00748	1.6173 ± 0.00496
Peak 2 Energy (eV)	2472.0	2471.9
Peak 2 Half-Width (eV)	0.75879 ± 0.00581	0.70139 ± 0.00433
Peak 3 Amplitude	0.81781 ± 0.0156	0.73056 ± 0.0201
Peak 3 Energy (eV)	2473.5	2473.1
Peak 3 Half-Width (eV)	0.85723 ± 0.0136	0.54795 ± 0.00713
Peak 4 Amplitude	0.9547 ± 0.0135	0.83558 ± 0.0151
Peak 4 Energy (eV)	2474.8	2474.0
Peak 4 Half-Width (eV)	0.89926 ± 0.0217	0.66103 ± 0.0229
Peak 5 Amplitude	-	0.71129 ± 0.0244
Peak 5 Energy (eV)	-	2474.7
Peak 5 Half-Width (eV)	-	0.47725 ± 0.0182

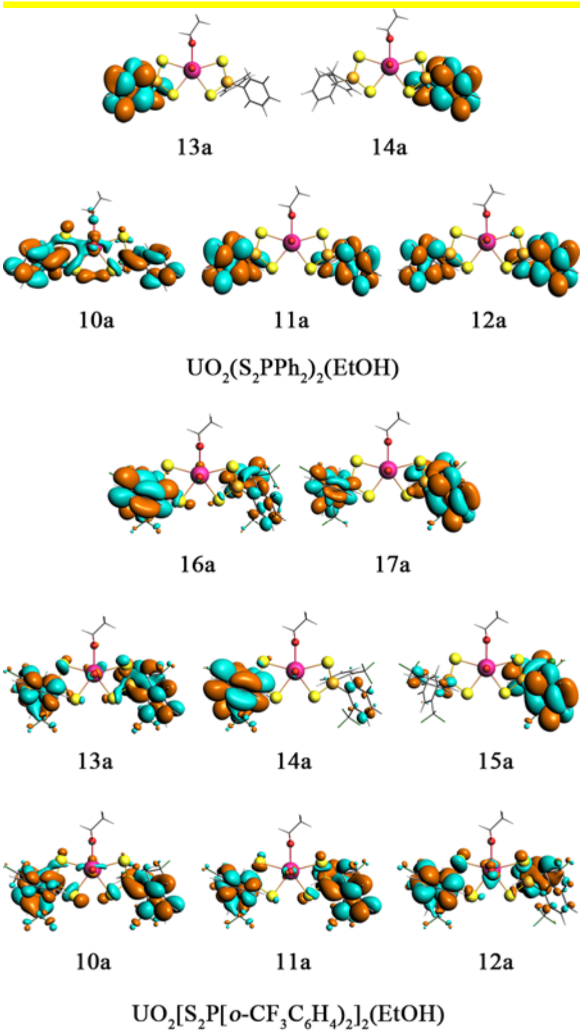

Figure S7 The contours of unoccupied Kohn-Sham orbitals with primarily phenyl character (C_π*) for $UO_2(S_2PR_2)_2(EtOH)$ in 0.02 au.
